# A word of caution regarding proposed benefits of albumin from ALBIOS: a dose of healthy skepticism

**DOI:** 10.1186/s13054-014-0509-x

**Published:** 2014-09-24

**Authors:** Alexander H Flannery, Sean P Kane, Angel O Coz-Yataco

**Affiliations:** Department of Pharmacy Services, University of Kentucky HealthCare, 800 Rose Street, H110, Lexington, KY 40536 USA; Department of Pharmacy Practice and Science, University of Kentucky College of Pharmacy, 800 Rose Street, H110, Lexington, KY 40536 USA; Department of Pharmacy Practice, Rosalind Franklin University of Medicine and Science, College of Pharmacy, Building: IPEC, Room: 2.803, 3333 Green Bay Road, North Chicago, IL 60064-3095 USA; Division of Pulmonary, Critical Care, and Sleep Medicine, University of Kentucky HealthCare, Kentucky Clinic, L543, 740S. Limestone, Lexington, KY 40536 USA

## Introduction

The recently published Albumin Italian Outcome Sepsis (ALBIOS) study was a prospective, open-label, multicenter, controlled trial of 1,818 patients with severe sepsis or septic shock [1]. Patients were randomized to receive either albumin 20% daily to maintain a serum albumin level ≥3 g/dl or no albumin replacement. No differences were detected in overall 28-day mortality, 90-day mortality, or a number of other secondary outcomes. However, three potential benefits of albumin were proposed that deserve further scrutiny.

## Caution in interpreting the subgroup analysis

A *post hoc*, exploratory analysis of patients with septic shock suggested a reduced relative risk of mortality at 90 days in the albumin group (relative risk 0.87; 95% confidence interval 0.77 to 0.99). While preserved when adjusting for imbalances in baseline characteristics, statistical significance was lost when adjusted for clinically relevant variables (relative risk 0.88; 95% confidence interval 0.77 to 1.01). More importantly, one must consider why a subgroup analysis of 90-day mortality, a secondary outcome, was performed rather than a subgroup analysis of the primary outcome, 28-day mortality. After all, 28-day mortality was the outcome reported in the hypothesis-generating Saline versus Albumin Fluid Evaluation study subgroup analysis of patients with severe sepsis [2]. The 28-day mortality comparison in the subgroup of patients with septic shock was presented by the ALBIOS study group at professional meetings, although not in the manuscript or appendix, and indeed is not significant (relative risk 0.95; 95% confidence interval 0.81 to 1.10) [3]. It is clinically difficult to explain a mortality benefit with albumin that only emerges beyond 28 days. Given the loss of statistical significance upon further regression analysis and the benefit seen only after 28 days, the small difference in 90-day mortality that initially emerged in the subgroup analysis of patients with septic shock is very likely due to chance.

## Caution in interpreting effect sizes

The ALBIOS trial suggested that albumin was associated with ‘small but significant hemodynamic advantages’, specifically a lower heart rate and higher mean arterial pressure [1]. In this analysis, statistical significance fails to represent clinical significance. The statistically significant differences in heart rate and mean arterial pressure ranged from 2 to 5 beats/minute and from 1 to 2 mm Hg, respectively. The lack of clinical significance is supported by the fact that meaningful differences failed to emerge at any time point in the central venous oxygen saturation and lactate values between the two groups. Similar claims of superiority are made for albumin with regard to net fluid balance. However, these differences were only statistically significant and marginally clinically significant on days 2 to 4, with the difference only favoring albumin by ≤300 ml/day.

## Caution in evaluating open-label endpoints at risk for bias

In the subgroup of patients with septic shock receiving vasopressors and/or inotropes at enrollment, a tertiary analysis suggested that use of albumin was associated with fewer days of vasopressor or inotrope support (median 3 days (interquartile range 1 to 6) vs. 4 days (interquartile range 2 to 7), *P* = 0.007). Of note, the ALBIOS trial was an open-label study, which may have biased the vasopressor titrations. Additionally, analyzing hours as opposed to days of vasopressor duration may have clarified the true effect size. Nevertheless, vasopressor agents are not benign medications and a reduction in their use could be of benefit.

## Implications of the ALBIOS trial

Aside from the benefit of potentially reducing vasopressor duration, the ALBIOS trial was largely a negative study for albumin in terms of improving outcomes compared with fluid alone. However, one must critically evaluate the data and published analysis or risk misinterpreting the study as favorable for albumin based on interpretations of the subgroup analysis and effect sizes. A multicenter French study investigating albumin in septic shock is expected to shed additional light on the relationship between albumin and vasopressor use [4].

Considering all available data, albumin should be considered a safe alternative to crystalloids. The cost-effectiveness of its use, however, remains a valid concern. Volume for volume, albumin is roughly 30 times more expensive than crystalloids [5]. Without any demonstrated superiority in clinical outcomes, it is difficult to justify extensive and unrestricted use of albumin at this time for resuscitation of patients with severe sepsis or septic shock.

## Abbreviation

ALBIOS: Albumin Italian Outcome Sepsis
